# Longitudinal Associations Among Workplace Social Capital, Career Calling and Work Engagement Among Nurses: A Three-Wave Cross-Lagged Panel Study

**DOI:** 10.3390/healthcare14203514

**Published:** 2026-10-19

**Authors:** Niuniu Sun, Yamei Liang, Yang Ni, Ruihong Zhang, Yu Xing, Fengsai Bie, Dongyu Song, Lin Cai

**Affiliations:** 1 School of Nursing, Henan University of Science and Technology, Luoyang 471000, China; 9905579@haust.edu.cn (N.S.); 240320332055@stu.haust.edu.cn (Y.L.); haustniyang@outlook.com (Y.N.); x2110677478@163.com (Y.X.); 2 Department of Nursing, the First Affiliated Hospital of Henan University of Science and Technology, Luoyang 471000, China; 3 Research and Education Department, Baoding No. 4 Central Hospital, Baoding 072350, China; 13931391449@163.com; 4 National Center for Occupational Safety and Health, National Health Commission of the People’s Republic of China, Beijing 102308, China; biefengsai0620@163.com; 5 Intensive Care Unit of Cardiac Surgery, Shandong Provincial Hospital Affiliated to Shandong First Medical University, Jinan 250021, China; songdongyu@sph.com.cn; 6 School of Education, Sichuan Institute of Industrial Technology, Deyang 618500, China

**Keywords:** social capital, career calling, work engagement, nurses, longitudinal studies, social cognitive theory

## Abstract

**Highlights:**

**What are the main findings?**

- Workplace social capital was prospectively associated with subsequent career calling and work engagement from T1 to T2 but not from T2 to T3, whereas work engagement showed significant longitudinal associations with subsequent workplace social capital across both intervals.
- Work engagement and career calling showed bidirectional longitudinal associations, suggesting that professional meaning and work engagement may be closely interconnected over time. Exploratory indirect-effect analyses identified several model-implied longitudinal indirect associations among the three constructs.

**What are the implications of the main findings?**

- The findings may inform the development of future interventions that consider workplace social capital, career calling, and work engagement as interconnected factors.
- The findings identify workplace social capital, career calling, and work engagement as potentially relevant factors for future longitudinal and intervention research.

**Abstract:**

**Background/Objectives**: Nurses experience substantial occupational challenges that may affect occupational well-being. Understanding longitudinal factors associated with occupational well-being is essential. This study examined the longitudinal associations among workplace social capital, career calling, and work engagement in nurses. **Methods**: A three-wave survey was conducted among 470 nurses recruited through convenience sampling from a single tertiary general hospital in China between March 2024 and March 2025. Workplace social capital, career calling, and work engagement were measured at six-month intervals using established self-report scales. Cross-lagged panel models were used to examine time-ordered associations and longitudinal indirect associations among the three variables, adjusting for covariates. **Results**: Workplace social capital, career calling, and work engagement showed temporal stability. Workplace social capital was prospectively associated with subsequent career calling and work engagement from T1 to T2 but not from T2 to T3. Career calling and work engagement showed significant longitudinal associations in both directions. Work engagement also showed longitudinal associations with later workplace social capital and career calling. Exploratory analyses identified several model-implied longitudinal indirect associations among workplace social capital, career calling, and work engagement over time. **Conclusions**: This study identified time-ordered longitudinal associations among workplace social capital, career calling and work engagement in nurses. Career calling and work engagement showed longitudinal associations in both directions, whereas workplace social capital was prospectively associated with subsequent career calling and work engagement mainly during the earlier interval. Because the traditional cross-lagged panel model (CLPM) does not distinguish within-person from between-person effects, these findings should be interpreted as longitudinal associations rather than evidence of causal or within-person reciprocal processes.

## 1. Introduction

Nurses play a pivotal role in healthcare systems by providing direct care, supporting patient recovery, promoting health and maintaining patient safety [1]. However, demanding work conditions may threaten nurses’ occupational well-being and workforce stability [2,3]. Sustaining nurses’ occupational well-being and professional motivation is therefore an important concern for healthcare managers and nursing leaders.

Work engagement is a key indicator of nurses’ positive occupational functioning. It refers to a positive, fulfilling, work-related state characterized by vigor, dedication and absorption [4]. Nurses with higher work engagement are more likely to show energy, persistence and commitment in clinical work. More broadly, favorable workforce well-being and supportive work environments have been associated with better care quality and patient safety [2,5,6]. Although the importance of work engagement has been widely recognized, less is known about how it develops and changes over time through the interaction of organizational and psychological factors.

Workplace social capital and career calling may be two important factors related to nurses’ work engagement. Workplace social capital reflects the quality of social relationships within the workplace, including trust, reciprocity, shared values, cooperation and supportive connections among colleagues and leaders [7,8,9]. In nursing settings, high workplace social capital may provide nurses with emotional support, timely information and practical assistance, which can help them cope with demanding clinical situations and maintain professional enthusiasm [10,11,12]. Career calling, by contrast, reflects the extent to which individuals experience their work as meaningful, purposeful and connected to a broader contribution to others or society [13]. For nurses, a sense of calling may be closely related to the professional mission of caring for patients and relieving suffering. More broadly, a stronger sense of calling has been associated with positive work-related outcomes across occupational contexts [14,15].

Although previous studies have examined associations among workplace social capital, career calling, and work engagement [1,10,11,14,15], the available evidence has largely been cross-sectional or has focused on individual constructs and related outcomes. To our knowledge, longitudinal evidence simultaneously examining all three constructs remains lacking, particularly among nurses. In addition, work engagement has often been treated primarily as a unidirectional outcome of workplace resources or professional meaning. Consequently, the dynamic interplay among nurses’ workplace social environment, professional meaning, and work-related behavior over time remains insufficiently understood.

**Figure 1:**
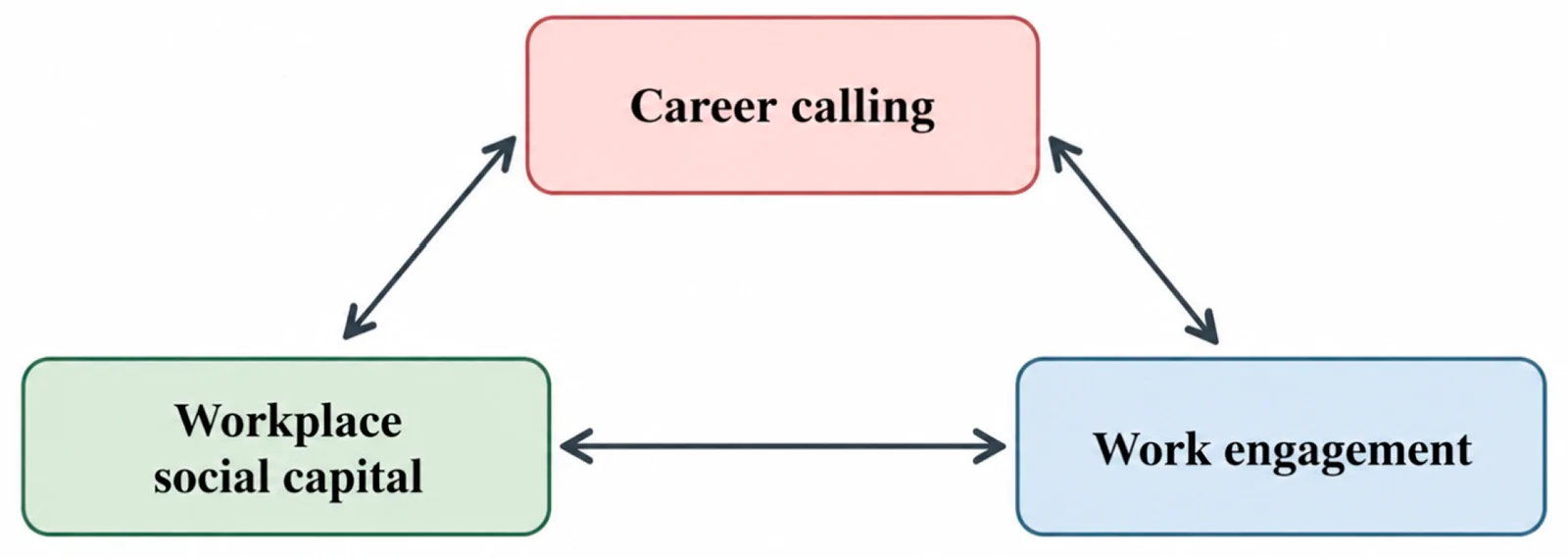
Conceptual framework of workplace social capital, career calling, and work engagement based on social cognitive theory.

Social cognitive theory provides a useful conceptual framework for considering this gap. According to Bandura’s triadic reciprocal determinism, human functioning is shaped through the continuous interaction of environmental conditions, personal cognitive factors and behavior [16]. In the present study, workplace social capital is conceptualized as a social-contextual workplace resource because it reflects the relational and organizational conditions perceived by nurses in their workplaces. Career calling is conceptualized as a personal cognitive and motivational factor because it captures nurses’ sense of professional meaning, purpose and mission. Work engagement is conceptualized as a positive work-related psychological state with behavioral manifestations, reflecting nurses’ energy, dedication and involvement in clinical work. Social cognitive theory was considered useful for the present study because its emphasis on interactions among contextual, personal, and behavioral factors provides a coherent perspective for examining longitudinal associations among workplace social capital, career calling, and work engagement. From this perspective, workplace social capital, career calling and work engagement may be understood as interrelated constructs within an environmental–personal–behavioral framework, making it relevant to examine their temporal ordering, bidirectional longitudinal associations, and longitudinal indirect associations within an integrated longitudinal model (Figure 1). In the present study, social cognitive theory was used as a conceptual lens rather than as a framework directly tested by cross-lagged panel model (CLPM).

Based on this theoretical framework, the present study proposes that workplace social capital, career calling, and work engagement are prospectively associated over time. Drawing on social cognitive theory, which highlights reciprocal interactions among environmental, personal, and behavioral factors, these relationships are expected to be dynamic rather than unidirectional. Accordingly, the following hypotheses are proposed:

**H1.**  *Workplace social capital and work engagement will show positive longitudinal associations in both directions over time.*

**H2.**  *Workplace social capital and career calling will show positive longitudinal associations in both directions over time.*

**H3.**  *Career calling and work engagement will show positive longitudinal associations in both directions over time.*

In addition to H1–H3, exploratory analyses were conducted to examine potential longitudinal indirect associations among workplace social capital, career calling and work engagement over time.

By testing these hypotheses, this study provides longitudinal evidence to clarify how workplace social capital, career calling and work engagement are dynamically connected among nurses.

## 2. Materials and Methods

### 2.1. Study Design and Setting

This study used a three-wave longitudinal observational design. Participants were recruited from a tertiary general hospital in Baoding, Hebei Province, China, and were drawn from more than 50 clinical and nursing-related units across the hospital. Data were collected at three time points at six-month intervals: March 2024 (T1), September 2024 (T2) and March 2025 (T3). Workplace social capital, career calling and work engagement were assessed at each wave using structured questionnaires. This study was reported in accordance with the Strengthening the Reporting of Observational Studies in Epidemiology (STROBE) guidelines.

### 2.2. Participants and Sampling

Participants were registered nurses working in the selected hospital. Convenience sampling was used. The nursing department and ward managers helped distribute study information to eligible nurses, but participation was voluntary.

The inclusion criteria were:

(1) Registered nurses with at least six months of clinical experience;
(2) Currently employed at the study hospital;
(3) Willingness to provide informed consent and participate in the survey.

The exclusion criteria were:

(1) Nurses who were on sick leave, maternity leave, external training or other forms of leave during the study period;
(2) Nurses who were unable to complete the electronic questionnaire independently because of physical limitations or other practical circumstances;
(3) Nurses who were participating in other similar research projects during the study period.

At the time of recruitment, 488 nurses met the eligibility criteria and were invited to participate in the study. Participation was voluntary, and nurses could decline participation without any consequences. Of these, 470 completed the baseline survey, corresponding to a baseline participation rate of 96.31%. Reasons for non-participation were not systematically recorded.

### 2.3. Data Collection Procedure

Data were collected using online structured questionnaires. Before each survey, participants were informed of the purpose of the study, the voluntary nature of participation and their right to withdraw at any time. Informed consent was obtained before questionnaire completion.

At T1, demographic information, workplace social capital, career calling and work engagement were collected. At T2 and T3, workplace social capital, career calling and work engagement were measured again. To match repeated responses while protecting participants’ privacy, anonymous identification codes were used across the three waves. Ethical approval was obtained before data collection, and all participants provided informed consent. The full ethical approval information is provided in the Institutional Review Board Statement.

### 2.4. Measures

#### 2.4.1. Demographic Information

Demographic and work-related characteristics were collected at baseline, including gender, age, highest educational qualification, marital status, professional title, years of work experience, leadership position, weekly working hours and average number of night shifts per week.

#### 2.4.2. Work Engagement

Work engagement was measured using the nine-item Utrecht Work Engagement Scale (UWES-9) developed by Schaufeli et al. [17]. In the present study, items were rated on a five-point Likert scale ranging from 1 (completely disagree) to 5 (completely agree). Previous Chinese studies, including research among nurses, have used the same five-point agreement response structure for the nine-item work-engagement measure [18,19], although the wording of the response anchors differed slightly. This response format was adopted at the questionnaire-design stage with reference to its prior use in Chinese work-engagement research. The scale includes three dimensions: vigor, dedication and absorption. Item scores were averaged to obtain the scale score, with higher scores indicating higher levels of work engagement. In the present study, Cronbach’s α values for the scale were 0.956 at T1, 0.935 at T2 and 0.948 at T3.

#### 2.4.3. Workplace Social Capital

Workplace social capital was measured using the Workplace Social Capital Scale developed by Kouvonen et al. [20]. The scale was adapted and validated for use in Chinese populations by Zhang et al. and has demonstrated good reliability in Chinese nurses [21,22]. The scale contains eight items covering cognitive and structural social capital. Items were rated on a five-point Likert scale ranging from 1 (completely disagree) to 5 (strongly agree). Item scores were averaged to obtain the scale score, with higher scores indicating higher workplace social capital. In the present study, Cronbach’s α values for the scale were 0.928 at T1, 0.930 at T2 and 0.926 at T3.

#### 2.4.4. Career Calling

Career calling was measured using the 12-item Calling Scale developed by Dobrow and Tosti-Kharas [23]. The Chinese version was adapted by Pei and Zhao and has subsequently demonstrated good reliability in Chinese nurses [24,25]. The scale is unidimensional and assesses the extent to which individuals experience their work as meaningful, purposeful and personally significant. Items were rated on a five-point Likert scale ranging from 1 (completely disagree) to 5 (completely agree). Item scores were averaged to obtain the scale score, with higher scores indicating a stronger sense of calling. In the present study, Cronbach’s α values were 0.963 at T1, 0.958 at T2 and 0.961 at T3.

### 2.5. Statistical Analysis

Data were analyzed using SPSS version 27.0 and Mplus version 8.3. SPSS was used to calculate descriptive statistics, including means, standard deviations and Pearson correlations for the study variables. Distributional assumptions and extreme outliers were examined before model estimation, and no serious violations were identified. All 470 participants who provided baseline data were included in the longitudinal analysis. Missing follow-up observations were handled in Mplus using full-information maximum likelihood (FIML) under maximum likelihood estimation. Baseline covariates were complete.

Potential common method variance was explored using complementary latent-variable diagnostics. First, an equality-constrained unmeasured latent method construct (ULMC) model was examined at each wave [26,27]. All observed items were specified to load on their respective substantive factors and on an additional latent method factor. The unstandardized method-factor loadings were constrained to a common freely estimated value across all items, the method factor was specified to be orthogonal to the substantive factors, and its variance was fixed at 1 for identification. Model admissibility, global model fit, and changes in standardized substantive factor loadings were examined. In addition, as a supplementary diagnostic, a single-factor confirmatory factor analysis (CFA) in which all 29 items loaded on one general factor was estimated at each wave and compared with the corresponding hypothesized three-factor measurement model [26,28].

Before testing the structural model, longitudinal measurement invariance was examined. Residuals of corresponding items were allowed to correlate across waves, and residual variances were constrained to equality when testing strict invariance. Mplus was then used to estimate a three-wave cross-lagged panel model to examine the longitudinal associations among workplace social capital, career calling and work engagement. The three constructs were specified as latent variables using their respective scale items as indicators. The model included autoregressive and cross-lagged paths between adjacent waves, as well as within-wave covariances among the three constructs. Model parameters were estimated using maximum likelihood (ML). Autoregressive and cross-lagged paths were freely estimated across the T1–T2 and T2–T3 intervals. The 12 cross-lagged paths corresponding to H1–H3 were treated as the primary hypothesis tests. The six Wald comparisons and the indirect-effect analyses were treated as secondary/exploratory analyses. Nominal *p*-values were reported for the primary analysis. As a sensitivity analysis for multiple testing, the Benjamini–Hochberg false discovery rate (FDR) procedure was applied across the 12 primary cross-lagged paths.

A Monte Carlo sensitivity analysis with 5000 replications was conducted to characterize the statistical sensitivity of the present sample for the simulated cross-lagged effects. With N = 470, 0.125 was the smallest tested cross-lagged coefficient yielding at least 80% power across all simulated cross-lagged paths (minimum power = 0.809). This sensitivity analysis should not be interpreted as demonstrating adequate statistical power for all parameters or hypothesis tests in the full longitudinal latent-variable model. Based on previous studies [29,30,31], age, marital status, professional title and leadership position were included as time-invariant baseline covariates. The four baseline covariates were specified to predict the latent constructs at each wave. As an additional sensitivity analysis, an expanded-covariate model further adjusted for baseline years of work experience, average working hours per week, and number of night shifts per week in addition to the four primary baseline covariates. An additional unadjusted CLPM excluding baseline covariates was estimated to compare the cross-lagged associations with those from the adjusted model. In addition, a cluster-robust model was estimated using robust maximum likelihood estimation with ward/unit membership specified as the clustering variable to account for potential within-unit dependency.

Indirect effects were tested using bootstrapping with 5000 resamples, and statistical significance was determined when the 95% confidence interval did not include zero. Temporal equality of corresponding unstandardized cross-lagged coefficients across the T1–T2 and T2–T3 intervals was formally evaluated using Wald tests. Model fit was evaluated using chi-square, the Comparative Fit Index (CFI), Tucker–Lewis Index (TLI), Root Mean Square Error of Approximation (RMSEA) [32], and Standardized Root Mean Square Residual (SRMR). Acceptable model fit was defined as CFI and TLI ≥ 0.90, RMSEA < 0.08, and SRMR < 0.08 [33].

## 3. Results

### 3.1. Characteristics of the Study Sample

A total of 470 nurses completed the baseline survey (T1), of whom 461 participated at T2 and 462 participated at T3. The three-wave data were matched using anonymous identification codes. All 470 participants who completed the baseline assessment were retained in the longitudinal analysis.

The average age of the 470 participants was 32.63 years (SD = 6.65), and 96.60% (*n* = 454) of the participants were female. More than half of the nurses held a bachelor’s degree or above, 75.74% (*n* = 356) were married, and 88.51% (*n* = 416) held junior or intermediate professional titles. Detailed characteristics are presented in Table 1.

### 3.2. Common Method Variance

The equality-constrained ULMC did not provide stable and consistently interpretable solutions across the three waves. At T1 and T2, the models yielded inadmissible solutions involving non-positive-definite latent covariance matrices, indicating that the method factor could not be stably separated from the substantive factors under this specification. Accordingly, parameter estimates from the T1 and T2 ULMC models were not interpreted. Although the T3 ULMC yielded an admissible solution, inclusion of the method factor materially attenuated several standardized substantive factor loadings, particularly those for workplace social capital. Therefore, the ULMC was not used to draw quantitative conclusions regarding the magnitude of common-method variance across waves.

As an additional diagnostic, single-factor CFA models were estimated at each wave. The single-factor models showed poor fit across T1–T3 (CFI = 0.642–0.679, TLI = 0.615–0.654, RMSEA = 0.143–0.147, SRMR = 0.117–0.129), whereas the corresponding hypothesized three-factor models showed substantially better fit (CFI = 0.962–0.969, TLI = 0.958–0.967, RMSEA = 0.046–0.049, SRMR = 0.027–0.034). These comparisons indicate that the covariance among the study items was not adequately represented by a single general factor. However, these diagnostic analyses do not establish the absence of common-method bias, and residual common-method and perceptual bias cannot be excluded. The model-fit comparisons are presented in Table 2.

### 3.3. Descriptive Analysis and Correlation Analysis

Table 3 presents the means, standard deviations, and correlations. Descriptively, workplace social capital, career calling, and work engagement showed modest fluctuations across the three waves. Mean levels generally increased from T1 to T2 and declined slightly at T3. Correlation analyses showed significant associations among workplace social capital, career calling, and work engagement both within and across waves, all in the expected direction.

### 3.4. Longitudinal Measurement Invariance

This study examined four levels of longitudinal measurement invariance across the three time points: the Configural invariance model, the Metric/Weak invariance model (equal factor loadings), the Scalar/Strong invariance model (equal item intercepts), and the strict invariance model (equal item residual variances). Measurement invariance was evaluated using changes in model fit indices, with |ΔCFI| < 0.01 and |ΔRMSEA| < 0.015 indicating acceptable invariance across increasingly constrained models [34]. As shown in Table 4, workplace social capital, career calling, and work engagement all met the criteria for strict longitudinal measurement invariance. Therefore, the measurement properties of all three constructs were considered sufficiently stable across the three waves for subsequent structural analyses. Standardized factor loadings for all items across the three waves are presented in Supplementary Table S1, ranging from 0.757–0.822 for workplace social capital, 0.791–0.846 for career calling, and 0.766–0.856 for work engagement.

### 3.5. Longitudinal Linkages Between Workplace Social Capital, Career Calling, and Work Engagement

Guided by social cognitive theory and the correlation results, a longitudinal cross-lagged panel model was employed to examine the temporal associations among workplace social capital, career calling, and work engagement. The model included autoregressive paths, cross-lagged paths and within-wave correlations among the three constructs. The model showed acceptable fit: χ^2^ = 5654.662, df = 3939, CFI = 0.951, TLI = 0.949, RMSEA = 0.030, 90% CI [0.029, 0.032], and SRMR = 0.031.

**Figure 2:**
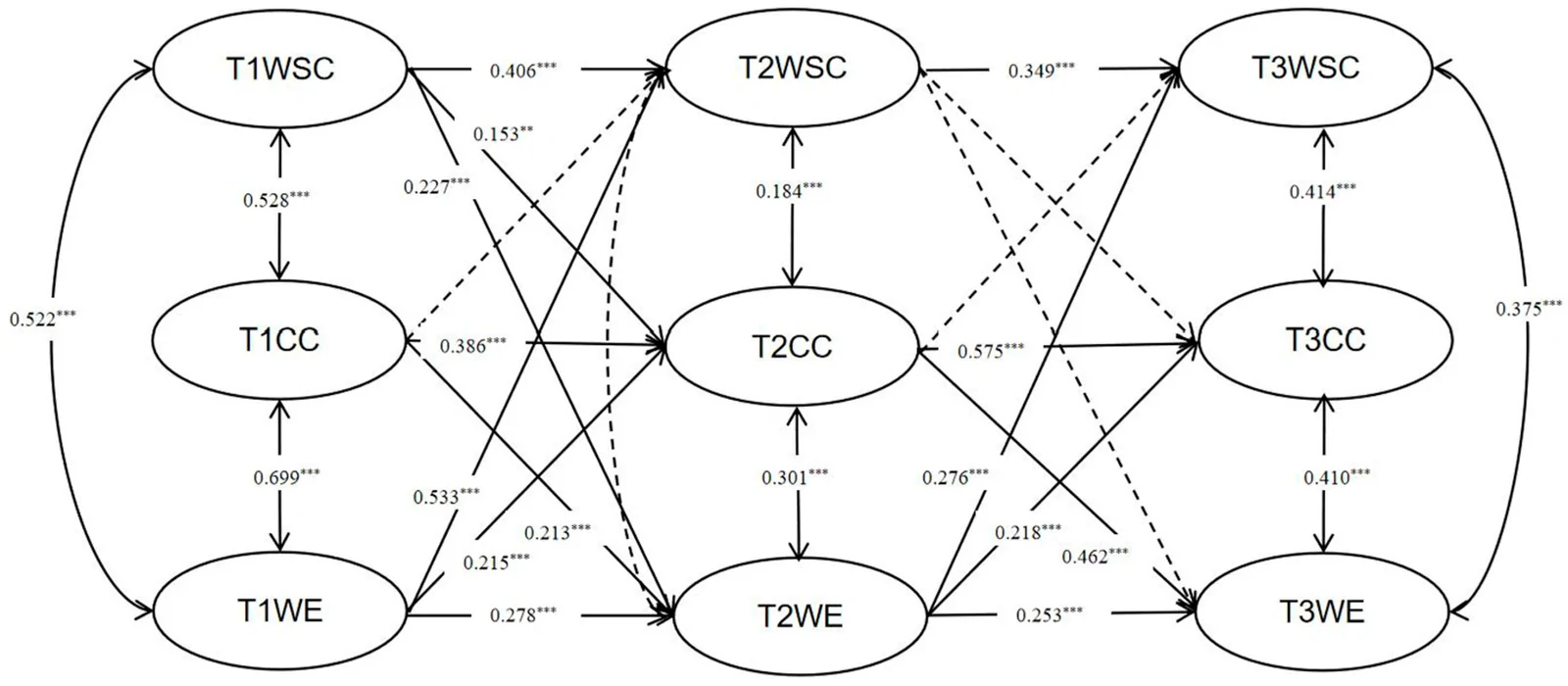
Cross-lagged panel model examining the longitudinal relationships among WSC, CC, and WE. Note: (1) WSC = workplace social capital; CC = career calling; WE = work engagement. (2) One-way arrows indicate directional autoregressive and cross-lagged paths; double-headed arrows indicate concurrent covariance among variables at the same time point; dashed lines indicate non-significant paths. (3) Demographic covariates, including nurses’ age, marital status, professional title and leadership position, are not shown to simplify the figure. (4) T1 = Time 1; T2 = Time 2; T3 = Time 3. (5) All the coefficients are standardized estimates. (6) ** *p* < 0.01; *** *p* < 0.001.

Figure 2 displays the final cross-lagged model, while detailed standardized estimates are presented in Table 5. The findings indicate time-ordered longitudinal associations among the three variables, some of which were observed in both directions. For workplace social capital and career calling, the cross-lagged path from T1 workplace social capital to T2 career calling was positive and significant (β = 0.153, *p* = 0.004), whereas the corresponding T2–T3 path was not significant (β = −0.054, *p* = 0.239). Neither reverse cross-lagged path from career calling to subsequent workplace social capital was significant (H2 partially supported). A similar temporal pattern was observed for workplace social capital and work engagement. The T1–T2 cross-lagged association was positive and significant (β = 0.227, *p* < 0.001), whereas the corresponding T2–T3 association was not significant (β = 0.024, *p* = 0.652). In contrast, work engagement showed significant positive cross-lagged associations with subsequent workplace social capital across both intervals (β = 0.533, *p* < 0.001; β = 0.276, *p* < 0.001) (H1 partially supported). Career calling and work engagement showed significant bidirectional longitudinal associations across both time intervals. Career calling showed positive cross-lagged associations with subsequent work engagement (β = 0.213, *p* < 0.001; β = 0.462, *p* < 0.001), while work engagement likewise showed positive cross-lagged associations with subsequent career calling (β = 0.215, *p* < 0.001; β = 0.218, *p* < 0.001). These findings were consistent with H3. The FDR-adjusted sensitivity analysis yielded the same inferential pattern, with all eight nominally significant cross-lagged paths remaining significant after FDR adjustment (Table 5). Both the unadjusted model and the expanded-covariate sensitivity model yielded the same pattern of direction and statistical significance across all 12 primary cross-lagged associations as the primary adjusted model (Supplementary Table S2). The cluster-robust sensitivity analysis included 55 wards/units, with cluster sizes ranging from 1 to 40 participants (median = 6). Standardized coefficients, robust standard errors, 95% confidence intervals, *p*-values, and model-fit indices are reported in Supplementary Table S3. The inferential pattern remained consistent with that of the primary model under the specified cluster correction.

As a secondary exploratory analysis, a Wald test was conducted to examine whether the corresponding cross-lagged coefficients differed between the T1–T2 and T2–T3 intervals. The overall test was significant, χ^2^ (6) = 32.384, *p* < 0.001. At the nominal *p*-value level, temporal differences were observed for WSC→CC (*p* = 0.002), CC→WE (*p* < 0.001), WSC→WE (*p* = 0.006), and WE→WSC (*p* = 0.011), whereas the differences for CC→WSC (*p* = 0.347) and WE→CC (*p* = 0.514) were not significant. Detailed comparisons are presented in Supplementary Table S4.

Furthermore, exploratory analyses identified several model-implied longitudinal indirect associations whose 95% bias-corrected bootstrap confidence intervals excluded zero (Table 6). Specifically, T1 workplace social capital and T1 work engagement were indirectly associated with T3 work engagement through T2 career calling (β = 0.071, *p* = 0.005; β = 0.099, *p* = 0.001). T1 workplace social capital and T1 career calling were indirectly associated with T3 career calling through T2 work engagement (β = 0.050, *p* = 0.002; β = 0.046, *p* = 0.005). In addition, T1 workplace social capital and T1 career calling were indirectly associated with T3 workplace social capital through T2 work engagement (β = 0.063, *p* = 0.001; β = 0.059, *p* = 0.005).

## 4. Discussion

The findings indicate that work engagement was prospectively associated with subsequent workplace social capital and career calling, suggesting that future longitudinal research should consider work engagement not only in relation to antecedent variables but also in relation to subsequent levels of other work-related constructs.

Overall, the longitudinal associations among workplace social capital, career calling, and work engagement were not uniformly bidirectional and were not temporally stable. Career calling and work engagement showed the most consistent bidirectional longitudinal associations, whereas the associations involving workplace social capital were more time-dependent. This pattern provides a basis for the following interpretation within the social cognitive framework.

The present study used a traditional cross-lagged panel model to examine the temporal relationships among workplace social capital, career calling and work engagement. Because this model does not fully separate within-person changes from stable between-person differences [35], the observed paths may reflect both time-ordered associations and enduring differences between nurses. Therefore, the findings should be interpreted as time-ordered longitudinal associations at the sample level rather than as evidence of causal relationships or purely within-person dynamics.

From a measurement perspective, work engagement and workplace social capital were modeled as global latent constructs; accordingly, the cross-lagged associations represent relationships between the overall constructs rather than their specific subdimensions. Given the mixed cross-sectional fit of the global-factor specifications, particularly the elevated RMSEA for work engagement at T3 (RMSEA = 0.105), these associations should be interpreted within the measurement structure adopted in this study.

### 4.1. Interpretation Through a Social Cognitive Perspective

The findings can be understood through a social cognitive perspective. According to this theory, human functioning is shaped by the interaction of environmental, personal and behavioral factors. In this study, workplace social capital was conceptualized as a social-contextual workplace resource, career calling as a personal cognitive and motivational factor, and work engagement as a positive work-related psychological state with behavioral manifestations. From this perspective, the observed longitudinal associations illustrate how these three constructs may be considered together within an environmental–personal–behavioral framework, rather than operating as isolated constructs.

However, the observed longitudinal associations were not consistently bidirectional or stable across both time intervals. This suggests that these associations may show asymmetric patterns across time in clinical settings. Environmental, personal and behavioral factors may be interrelated, but the strength and direction of these associations may vary across time and work contexts.

### 4.2. Workplace Social Capital as an Environmental Foundation

Workplace social capital was prospectively associated with subsequent career calling and work engagement from T1 to T2. This finding suggests that a trusting and cooperative workplace may represent an important social-contextual resource related to nurses’ professional meaning and engagement. In clinical nursing settings, social capital may be reflected in mutual trust, shared goals, reciprocity, communication and supportive leadership [5,10]. These social conditions may help nurses obtain emotional support, information and practical help. They may also reduce feelings of isolation and increase nurses’ sense of belonging.

In complex and demanding clinical environments, social support and cooperation may help nurses maintain professional meaning and invest energy in patient care. Recent nursing research has similarly highlighted the importance of the work environment and social support for nurses’ well-being, work engagement and retention [5]. Therefore, workplace social capital may represent an important contextual factor associated with nurses’ professional motivation and work engagement.

The cross-lagged associations of workplace social capital with subsequent career calling and work engagement were not significant from T2 to T3. Exploratory Wald tests indicated that the WSC→CC and WSC→WE coefficients differed between the T1–T2 and T2–T3 intervals at the nominal *p*-value level (*p* = 0.002 and *p* = 0.006, respectively), indicating temporal heterogeneity in these associations. The non-significant T2–T3 paths indicate that the associations between workplace social capital and later career calling and work engagement were less evident during the later interval. However, the reasons for these temporal differences cannot be determined from the available data. Future studies with more measurement waves and time-varying organizational or contextual information are needed to clarify these temporal patterns.

### 4.3. Bidirectional Longitudinal Associations Between Career Calling and Work Engagement

A central finding of this study was that career calling and work engagement showed significant longitudinal associations in both directions. Career calling was prospectively associated with subsequent work engagement, and work engagement was also prospectively associated with subsequent career calling. This pattern extends previous evidence linking career calling with positive work-related outcomes by showing that the relationship was evident longitudinally in both directions.

Career calling reflects the perceived purpose, value and social significance of one’s work, whereas work engagement reflects a positive work-related state characterized by vigor, dedication and absorption [13,17,36,37]. The observed association from career calling to subsequent work engagement is therefore consistent with the conceptual linkage between professional meaning and work involvement. Conversely, the association from work engagement to subsequent career calling indicates that the longitudinal relationship was not confined to a single temporal direction. Recent studies have similarly linked calling with positive work-related outcomes, including work engagement and safety behavior among nurses [31,38]. Overall, these findings indicate bidirectional time-ordered associations between career calling and work engagement at the sample level, while the mechanisms underlying these associations were not examined in the present study.

### 4.4. Longitudinal Associations of Work Engagement with Subsequent Workplace Social Capital and Career Calling

Work engagement may be prospectively associated with subsequent perceptions of workplace social capital. Nurses with higher work engagement may subsequently perceive greater trust, reciprocity, cooperation and support within their work environment. However, because workplace social capital was measured by self-report, these findings should not be interpreted as evidence that work engagement directly changed team-level relationships or organizational social capital.

Work engagement was also prospectively associated with subsequent career calling across both time intervals. More broadly, nursing research has shown associations between work motivation and work engagement [37]. As this evidence is cross-sectional, it provides contextual support for associations between motivational and engagement-related constructs rather than evidence for the temporal direction or mechanisms observed in the present study. The associations observed here remained evident after accounting for prior levels of career calling and the included covariates. These findings further indicate that work engagement was prospectively associated with both subsequent perceived workplace social capital and career calling.

### 4.5. Model-Implied Longitudinal Indirect Associations

Exploratory analyses identified several model-implied longitudinal indirect associations among workplace social capital, career calling, and work engagement over time. Specifically, workplace social capital at T1 and work engagement at T1 were indirectly associated with work engagement at T3 through career calling at T2. In addition, workplace social capital at T1 and career calling at T1 were indirectly associated with career calling at T3 and workplace social capital at T3 through work engagement at T2.

These model-implied indirect associations describe the statistical ordering of the three constructs across the measurement waves. Overall, the indirect effects were modest in magnitude and should be interpreted as exploratory time-ordered statistical associations rather than evidence of causal mediation or specific underlying mechanisms. As model-implied estimates from observational data, they also remain subject to model specification and potential unmeasured confounding.

### 4.6. Implications for Practice and Policy

The findings highlight the potential relevance of workplace relationships, professional meaning and work engagement for future nursing workforce interventions. These factors may therefore warrant further evaluation as potential targets in subsequent longitudinal and intervention studies.

Future studies could evaluate whether organizational or professional-development interventions targeting relational workplace resources, professional meaning or work engagement lead to beneficial changes over time. Specific approaches, such as peer support, mentoring or professional reflection, should be regarded as hypotheses for future intervention research rather than evidence-based recommendations arising from the present study.

### 4.7. Strengths, Limitations and Future Research

This study has several strengths. First, guided by social cognitive theory, it integrated workplace social capital, career calling and work engagement into a single environmental–personal–behavioral framework. Second, the three-wave longitudinal design allowed the study to examine temporal ordering, bidirectional cross-lagged associations, and exploratory longitudinal indirect associations, providing evidence beyond cross-sectional associations. Third, the study achieved a high retention rate across the three survey waves.

Several limitations should also be acknowledged. First, all main variables were measured using self-report questionnaires, which may introduce response bias and inflate the observed associations because of shared method variance. Although potential common method variance was explored using ULMC and single-factor CFA diagnostics, the ULMC did not yield stable and consistently interpretable solutions across waves. At T3, inclusion of the method factor substantially attenuated several substantive factor loadings, suggesting that shared measurement effects may have influenced the observed associations. These post hoc diagnostic analyses cannot rule out common-method bias. Residual self-report, same-source, and perceptual bias therefore remain possible. Future studies could incorporate supervisor ratings, administrative data or team-level indicators. In addition, the five-point agreement format used for work engagement may introduce some uncertainty regarding measurement validity and limit direct comparability with studies using other UWES response formats. Second, participants were recruited using convenience sampling from a single tertiary general hospital in China, and the sample was predominantly female, which may limit the generalizability of the findings to other healthcare settings, geographic regions and more diverse nursing populations. Future studies should recruit larger, multicenter and more diverse samples. Third, ward/unit membership was incorporated into a cluster-robust sensitivity analysis. Although the inferential pattern of the main cross-lagged associations remained unchanged under the cluster-robust correction for within-unit dependency, this analysis did not explicitly estimate unit-level random effects or between-unit variation. Future studies with larger and more balanced samples across units could further examine potential unit-level influences using multilevel longitudinal models. Fourth, the traditional cross-lagged panel model used in this study does not distinguish within-person fluctuations from stable between-person differences. Although an additional sensitivity model further adjusted for baseline work experience, working hours, and night shifts, potential time-varying confounding arising from changes in work schedules or other occupational conditions across the follow-up period was not modeled. This represents an additional limitation to causal interpretation of the observed cross-lagged associations. We also attempted an a random-intercept cross-lagged panel model (RI-CLPM) as a sensitivity analysis; however, the model produced a non-positive-definite latent covariance matrix with negative random-intercept variances and was therefore not interpreted. Future studies using RI-CLPM or related approaches, ideally with additional measurement waves and larger samples, are needed to clarify within-person longitudinal dynamics. In addition, the six-month measurement interval was prespecified but should not be regarded as an established optimal lag; studies using alternative intervals and additional measurement waves may help clarify the timing of these longitudinal associations. Finally, qualitative, mixed-methods and intervention studies are needed to further clarify how workplace social capital and career calling are longitudinally related to nurses’ work engagement over time.

## 5. Conclusions

This three-wave longitudinal study identified time-ordered longitudinal associations among workplace social capital, career calling and work engagement in nurses. Career calling and work engagement showed significant longitudinal associations in both directions, whereas workplace social capital was prospectively associated with later career calling and work engagement mainly during the earlier time interval. Work engagement also showed longitudinal associations with subsequent workplace social capital and career calling. Because the traditional CLPM does not distinguish within-person from between-person effects, these findings should not be interpreted as evidence of within-person reciprocal processes or mutual reinforcement. Given the single-center convenience sample, predominance of female participants, self-reported measures, and the possibility of residual time-varying confounding, the findings should be interpreted cautiously. Further multicenter longitudinal studies using analytical approaches capable of separating within-person and between-person effects are needed to clarify these associations.

## Figures and Tables

**Table 1:** Demographic characteristics of participants (N = 470).

Variable	Category	N (%)
Gender	Male	16 (3.40)
	Female	454 (96.60)
Age (years), Mean (SD)		32.63 (6.65)
Education	Below bachelor’s degree	146 (31.06)
	Bachelor’s degree or above	324 (68.94)
Marital status	Not married	114 (24.26)
	Married	356 (75.74)
Professional title	Junior	238 (50.64)
	Intermediate	178 (37.87)
	Senior	54 (11.49)
Years of work experience	0–5	132 (28.09)
	6–10	81 (17.23)
	11–15	172 (36.60)
	16–20	45 (9.57)
	>20	40 (8.51)
Leadership position	Yes	75 (15.96)
	No	395 (84.04)
Average working hours per week	≤40 h	64 (13.62)
	41–50 h	189 (40.21)
	51–60 h	126 (26.81)
	61–70 h	57 (12.13)
	>70 h	34 (7.23)
Night shifts per week	0	134 (28.51)
	1	130 (27.66)
	2	125 (26.60)
	3	25 (5.32)
	≥4	56 (11.91)

Note: (1) Percentages may not add up to 100% owing to rounding. (2) Abbreviation: SD, standard deviation. (3) “Not married” included participants who were never married, divorced, widowed, or separated.

**Table 2:** Comparison of single-factor and hypothesized three-factor measurement models across waves.

Wave	Model	χ^2^(df)	CFI	TLI	RMSEA	SRMR
T1	Single-factor	4202.110 (377)	0.679	0.654	0.147	0.119
	Three-factor	739.460 (374)	0.969	0.967	0.046	0.027
T2	Single-factor	4060.271 (377)	0.642	0.615	0.145	0.129
	Three-factor	768.883 (374)	0.962	0.958	0.048	0.034
T3	Single-factor	3958.026 (377)	0.677	0.652	0.143	0.117
	Three-factor	798.397 (374)	0.962	0.958	0.049	0.031

Note: The single-factor CFA was used as a supplementary diagnostic to examine whether the covariance among all study items could be adequately represented by a single general factor; it should not be interpreted as demonstrating the absence of common-method bias.

**Table 3:** Descriptive statistics and correlations among study variables (N = 470).

	Mean (SD)	1	2	3	4	5	6	7	8	9
1. T1WSC	4.036 (0.711)	--								
2. T2WSC	4.148 (0.681)	0.605 ***	--							
3. T3WSC	4.123 (0.678)	0.326 ***	0.463 ***	--						
4. T1CC	3.689 (0.813)	0.497 ***	0.508 ***	0.316 ***	--					
5. T2CC	3.854 (0.762)	0.445 ***	0.515 ***	0.350 ***	0.597 ***	--				
6. T3CC	3.748 (0.784)	0.324 ***	0.352 ***	0.510 ***	0.406 ***	0.645 ***	--			
7. T1WE	3.599 (0.860)	0.490 ***	0.666 ***	0.417 ***	0.670 ***	0.544 ***	0.369 ***	--		
8. T2WE	3.826 (0.725)	0.445 ***	0.473 ***	0.432 ***	0.503 ***	0.544 ***	0.490 ***	0.514 ***	--	
9. T3WE	3.659 (0.789)	0.294 ***	0.387 ***	0.504 ***	0.390 ***	0.594 ***	0.644 ***	0.395 ***	0.498 ***	--

Note: (1) WSC = workplace social capital; CC = career calling; WE = work engagement; (2) T1 = Time 1; T2 = Time 2; T3 = Time 3; (3) *** *p* < 0.001.

**Table 4:** Longitudinal measurement invariance model fit indices.

Construct	Model	Model Fit Indices						Model Comparison		
		χ^2^	df	CFI	TLI	RMSEA	SRMR	Comparison	ΔCFI	ΔRMSEA
WSC	M1	507.924	225	0.963	0.955	0.052	0.033			
	M2	520.388	239	0.963	0.958	0.050	0.040	M2 − M1	0.000	−0.002
	M3	536.420	253	0.963	0.960	0.049	0.042	M3 − M2	0.000	−0.001
	M4	557.239	269	0.962	0.961	0.048	0.047	M4 − M3	−0.001	−0.001
CC	M1	1145.333	555	0.961	0.956	0.048	0.029			
	M2	1156.981	577	0.962	0.958	0.046	0.032	M2 − M1	0.001	−0.002
	M3	1187.422	599	0.961	0.959	0.046	0.033	M3 − M2	−0.001	0.000
	M4	1215.074	623	0.961	0.961	0.045	0.036	M4 − M3	0.000	−0.001
WE	M1	644.474	294	0.966	0.960	0.050	0.029			
	M2	651.700	310	0.967	0.963	0.048	0.031	M2 − M1	0.001	−0.002
	M3	671.632	326	0.967	0.964	0.047	0.032	M3 − M2	0.000	−0.001
	M4	692.261	342	0.966	0.965	0.047	0.035	M4 − M3	−0.001	−0.001

Note: (1) WSC = workplace social capital; CC = career calling; WE = work engagement; (2) M1 = configural invariance model; M2 = metric invariance model; M3 = scalar invariance model; M4 = strict invariance model.

**Table 5:** Standardized estimates, standard errors, confidence intervals, and nominal and FDR-adjusted *p*-values for the primary cross-lagged paths.

Path	β	SE	95% CI	Nominal *p*	FDR-Adjusted *p*
T1 WSC → T2 CC	0.153	0.054	[0.052, 0.260]	0.004	0.006
T2 WSC → T3 CC	−0.054	0.046	[−0.142, 0.039]	0.239	0.319
T1 CC → T2 WSC	−0.052	0.052	[−0.156, 0.050]	0.322	0.386
T2 CC → T3 WSC	0.023	0.056	[−0.090, 0.134]	0.689	0.689
T1 WSC → T2 WE	0.227	0.053	[0.123, 0.333]	<0.001	<0.001
T2 WSC → T3 WE	0.024	0.053	[−0.076, 0.129]	0.652	0.689
T1 WE → T2 WSC	0.533	0.052	[0.431, 0.635]	<0.001	<0.001
T2 WE → T3 WSC	0.276	0.058	[0.159, 0.390]	<0.001	<0.001
T1 CC → T2 WE	0.213	0.060	[0.096, 0.336]	<0.001	<0.001
T2 CC → T3 WE	0.462	0.050	[0.362, 0.559]	<0.001	<0.001
T1 WE → T2 CC	0.215	0.059	[0.101, 0.332]	<0.001	<0.001
T2 WE → T3 CC	0.218	0.048	[0.124, 0.312]	<0.001	<0.001

Note: (1) WSC = workplace social capital; CC = career calling; WE = work engagement. (2) β = STDYX standardized regression coefficient; SE = standard error; CI = bias-corrected bootstrap confidence interval based on 5000 resamples. (3) All estimates were adjusted for age, marital status, professional title, and leadership position. (4) FDR-adjusted *p*-values were calculated across the 12 primary cross-lagged paths using the Benjamini–Hochberg procedure.

**Table 6:** Standardized longitudinal indirect effects among WSC, CC and WE.

Indirect Paths	Estimated Value of the Standardized Indirect Effect	*p*	Standardized 95%BC Bootstrap CI	
			Lower Limit	Upper Limit
T1WSC → T2CC → T3WE	0.071	0.005	0.025	0.122
T1WE → T2CC → T3WE	0.099	0.001	0.045	0.168
T1WSC → T2WE → T3CC	0.050	0.002	0.024	0.088
T1CC → T2WE → T3CC	0.046	0.005	0.020	0.089
T1WSC → T2WE → T3WSC	0.063	0.001	0.031	0.109
T1CC → T2WE → T3WSC	0.059	0.005	0.025	0.109

Note: (1) WSC = workplace social capital; CC = career calling; WE = work engagement. (2) T1 = Time 1; T2 = Time 2; T3 = Time 3. (3) The indirect-effect analyses were exploratory, and the reported *p*-values were not adjusted for multiple testing.

## Data Availability

The data that support the findings of this study are available from the corresponding author upon reasonable request. The data are not publicly available due to privacy and ethical restrictions related to participant confidentiality.

## Supplementary Materials

The following supporting information can be downloaded at: https://www.mdpi.com/article/10.3390/healthcare14203514/s1, Table S1: Standardized factor loadings of scale items across the three waves; Table S2: Comparison of standardized cross-lagged estimates across unadjusted, primary adjusted, and expanded-covariate models; Table S3: Comparison of standardized cross-lagged estimates between the primary and cluster-robust models; Table S4: Wald tests comparing cross-lagged coefficients across time intervals.
