# Supplementary material for: Longitudinal Associations Among Workplace Social Capital, Career Calling and Work Engagement Among Nurses: A Three-Wave Cross-Lagged Panel Study
Source: Healthcare (Basel). 2026 Oct 19;14(20):3514. doi: 10.3390/healthcare14203514 (PMC13704150; doi:10.3390/healthcare14203514)
Supplement: Supplementary file 1 [file healthcare-14-03514-s001.zip › healthcare-4447074-supplementary.pdf]

Supplementary Table S1. Standardized factor loadings of scale items across the three waves

Construct	Item	T1	T2	T3
Workplace social capital	WSC1	0.772	0.810	0.788
	WSC2	0.757	0.775	0.762
	WSC3	0.763	0.780	0.775
	WSC4	0.785	0.778	0.822
	WSC5	0.797	0.801	0.759
	WSC6	0.804	0.798	0.774
	WSC7	0.815	0.794	0.803
	WSC8	0.792	0.780	0.761
Career calling	CC1	0.830	0.808	0.835
	CC2	0.831	0.798	0.834
	CC3	0.846	0.808	0.807
	CC4	0.818	0.816	0.817
	CC5	0.830	0.791	0.819
	CC6	0.821	0.803	0.821
	CC7	0.844	0.830	0.819
	CC8	0.801	0.827	0.819
	CC9	0.834	0.821	0.805
	CC10	0.826	0.806	0.819
	CC11	0.834	0.802	0.820
	CC12	0.830	0.800	0.835
Work engagement	WE1	0.844	0.808	0.823
	WE2	0.837	0.785	0.855
	WE3	0.831	0.779	0.822
	WE4	0.843	0.774	0.825
	WE5	0.848	0.783	0.812
	WE6	0.824	0.766	0.776
	WE7	0.856	0.796	0.804
	WE8	0.847	0.793	0.831
	WE9	0.848	0.781	0.809

Note: (1) WSC = workplace social capital; CC = career calling; WE = work engagement; T1 = Time 1; T2 = Time 2; T3 = Time 3. (2) Values are STDYX-standardized factor loadings estimated from the configural longitudinal measurement models.

Supplementary Table S2. Comparison of standardized cross-lagged estimates across unadjusted, primary adjusted, and expanded-covariate models

Path	Unadjusted model β (p)	Primary adjusted model β (p)	Expanded-covariate model β (p)
T1 WSC → T2 CC	0.152 (0.001)	0.153 (0.004)	0.153 (0.001)
T2 WSC → T3 CC	−0.050 (0.286)	−0.054 (0.239)	−0.053 (0.252)
T1 CC → T2 WSC	−0.052 (0.297)	−0.052 (0.322)	−0.050 (0.321)

Path	Unadjusted model<br>β (p)	Primary adjusted model<br>β (p)	Expanded-covariate model<br>β (p)
T2 CC → T3 WSC	0.023 (0.682)	0.023 (0.689)	0.017 (0.761)
T1 WSC → T2 WE	0.225 (<0.001)	0.227 (<0.001)	0.219 (<0.001)
T2 WSC → T3 WE	0.029 (0.553)	0.024 (0.652)	0.025 (0.611)
T1 WE → T2 WSC	0.535 (<0.001)	0.533 (<0.001)	0.531 (<0.001)
T2 WE → T3 WSC	0.272 (<0.001)	0.276 (<0.001)	0.286 (<0.001)
T1 CC → T2 WE	0.218 (<0.001)	0.213 (<0.001)	0.211 (<0.001)
T2 CC → T3 WE	0.462 (<0.001)	0.462 (<0.001)	0.459 (<0.001)
T1 WE → T2 CC	0.214 (<0.001)	0.215 (<0.001)	0.214 (<0.001)
T2 WE → T3 CC	0.208 (<0.001)	0.218 (<0.001)	0.225 (<0.001)

Note: (1) WSC = workplace social capital; CC = career calling; WE = work engagement; T1 = Time 1; T2 = Time 2; T3 = Time 3; (2) *β* = STDYX standardized regression coefficient; (3) The unadjusted model included no baseline covariates. (4) The primary adjusted model controlled for age, marital status, professional title, and leadership position. (5) The expanded-covariate model additionally controlled for baseline years of work experience, average working hours per week, and number of night shifts per week.

Supplementary Table S3. Comparison of standardized cross-lagged estimates between the primary and cluster-robust models

Path	Primary model β	Primary model p	Cluster-robust β	Robust SE	95% CI	Robust p
T1 WSC → T2 CC	0.153	0.004	0.153	0.052	[0.052, 0.254]	0.003
T2 WSC → T3 CC	−0.054	0.239	−0.054	0.044	[−0.140, 0.032]	0.218
T1 CC → T2 WSC	−0.052	0.322	−0.052	0.049	[−0.148, 0.044]	0.292
T2 CC → T3 WSC	0.023	0.689	0.023	0.058	[−0.092, 0.137]	0.698
T1 WSC → T2 WE	0.227	<0.001	0.227	0.040	[0.149, 0.306]	<0.001
T2 WSC → T3 WE	0.024	0.652	0.024	0.056	[−0.087, 0.134]	0.674
T1 WE → T2 WSC	0.533	<0.001	0.533	0.048	[0.438, 0.628]	<0.001
T2 WE → T3 WSC	0.276	<0.001	0.276	0.059	[0.161, 0.392]	<0.001
T1 CC → T2 WE	0.213	<0.001	0.213	0.045	[0.125, 0.301]	<0.001
T2 CC → T3 WE	0.462	<0.001	0.462	0.054	[0.356, 0.567]	<0.001

Path	Primary model β	Primary model p	Cluster-robust β	Robust SE	95% CI	Robust p
T1 WE → T2 CC	0.215	<0.001	0.215	0.058	[0.101, 0.328]	<0.001
T2 WE → T3 CC	0.218	<0.001	0.218	0.043	[0.134, 0.303]	<0.001

Note: (1) WSC = workplace social capital; CC = career calling; WE = work engagement; T1 = Time 1; T2 = Time 2; T3 = Time 3; β = STDYX standardized regression coefficient; SE = standard error; CI = confidence interval. (2) The primary model was adjusted for age, marital status, professional title, and leadership position. (3) The cluster-robust model specified ward/unit membership as the clustering variable and used robust maximum likelihood estimation. (4) The cluster-robust analysis included 55 wards/units; cluster sizes ranged from 1 to 40 participants, with a median of 6 participants per unit. The cluster-size distribution was as follows: 10 units included 1 participant, 5 included 2, 5 included 3, 1 included 4, 2 included 5, 9 included 6, 4 included 7, 3 included 8, 2 included 11, 5 included 12, 1 included 16, 3 included 21, 1 included 24, 2 included 26, 1 included 38, and 1 included 40 participants. (5) The cluster-robust model showed the following fit: $\chi^2(3939) = 5708.416$, CFI = 0.949, TLI = 0.947, RMSEA = 0.031 (90% CI [0.029, 0.033]), and SRMR = 0.031.

Supplementary Table S4. Wald tests comparing cross-lagged coefficients across time intervals

Cross-lagged path	T1→T2 B	T2→T3 B	Difference in B	p
WSC→CC	0.159	−0.060	0.219	0.002
CC→WSC	−0.043	0.022	−0.064	0.347
CC→WE	0.191	0.503	−0.312	<0.001
WE→CC	0.178	0.222	−0.044	0.514
WSC→WE	0.247	0.027	0.220	0.006
WE→WSC	0.425	0.252	0.173	0.011

Note: WSC = workplace social capital; CC = career calling; WE = work engagement; B = unstandardized regression coefficient.
